# Community Structure, Growth-Promoting Potential, and Genomic Analysis of Seed-Endophytic Bacteria in *Stipagrostis pennata*

**DOI:** 10.3390/microorganisms13081754

**Published:** 2025-07-27

**Authors:** Yuanyuan Yuan, Shuyue Pang, Wenkang Niu, Tingting Zhang, Lei Ma

**Affiliations:** 1 College of Life Science, Shihezi University, Shihezi 832000, China; yuanyuan_yuan1128@163.com (Y.Y.); m18235295280@163.com (S.P.); wenkang_niu@163.com (W.N.); 2 Key Laboratory of Oasis Town and Mountain-Basin System Ecology of Bingtuan, Shihezi University, Shihezi 832000, China

**Keywords:** endophytic microbiota, plant–microbe interaction, stress tolerance mechanisms, microbial biostimulant, desert plant adaptation, functional genomics

## Abstract

*Stipagrostis pennata* is an important plant in desert ecosystems. Its seed-endophytic bacteria may play a critical role in plant growth and environmental adaptation processes. This study systematically analyzed the community composition and potential plant growth-promoting (PGP) functions of seed-endophytic bacteria associated with *S. pennata*. The results showed that while the overall diversity of bacterial communities from different sampling sites was similar, significant differences were observed in specific functional genes and species abundances. Nine endophytic bacterial strains were isolated from the seeds, among which *Bacillus altitudinis* strain L7 exhibited phosphorus solubilizing capabilities, nitrogen fixing, IAA production, siderophore generation, and multi-hydrolytic enzyme activities. Additionally, the genomic sequencing of L7 revealed the key genes involved in plant growth promotion and environmental adaptation, including Na^+^ efflux systems, K^+^ transport systems, compatible solute synthesis genes, and the gene clusters associated with nitrogen metabolism, IAA synthesis, phosphate solubilization, and siderophore synthesis. Strain L7 exhibits salt and osmotic stress tolerance while promoting plant growth, providing a promising candidate for desert microbial resource utilization and plant biostimulant development.

## 1. Introduction

*Stipagrostis pennata* is a perennial herbaceous species belonging to the genus Stipagrostis in the Poaceae family [1]. It exhibits excellent characteristics such as sand burial tolerance, wind erosion resistance, and salt–alkali tolerance, making it a pioneering plant for stabilizing shifting sands and improving the micro-environment of sandy lands in deserts [2].

Seed endophytes refer to microorganisms that live inside seeds, forming symbiotic or coexistent relationships with the host without causing disease [3]. Seed endophytes can directly or indirectly promote plant growth through functions such as producing plant hormones, fixing nitrogen, solubilizing phosphates, and producing siderophores [4,5,6,7]. Additionally, they can enhance the stress resistance of host plants by mechanisms including increasing the content of osmotic regulators like proline, betaine, and glycine within the host plant [8,9], boosting antioxidant enzyme activity [10,11], and reducing malondialdehyde (MDA) content [12].

However, the community structure, growth-promoting potential, and mechanisms of seed endophytes in *S. pennata* remain unclear. Therefore, this study analyzed the diversity and community composition of seed-endophytic bacteria in *S. pennata* using 16S rRNA amplicon sequencing. Subsequently, functional strains were isolated, and growth promotion experiments and genomic analysis were conducted. This research provides a scientific basis for the screening and application of seed endophytes in *S. pennata*.

## 2. Materials and Methods

### 2.1. Material Pretreatment

The seeds of *S. pennata* used in the experiment were collected from the hinterland (A) and southern edge (B) of the Gurbantunggut Desert. Seed surface sterilization was performed with modifications based on reference [13]. The seeds were rinsed three times with sterile distilled water (5 min each), and then surface moisture was blotted dry with sterile filter paper. Subsequently, the seeds were immersed in 75% ethanol for 1 min, rinsed with sterile distilled water, immersed in 3% sodium hypochlorite solution for 3 min, rinsed again with sterile distilled water, immersed in 75% ethanol for 30 s, and finally soaked in sterile distilled water for 5 min followed by repeated rinsing. A 150 μL aliquot of the final rinse was spread on LB agar plates and incubated at 30 °C for 2~5 days. The absence of colony growth confirmed the thorough disinfection.

### 2.2. Total DNA Extraction, PCR Amplification, and High-Throughput Sequencing

Total DNA extraction was performed using the E.Z.N.A.^®^ Soil DNA Kit (Omega Bio-Tek, Norcross, GA, USA). After extraction, the genomic DNA was examined via 1% agarose gel electrophoresis. PCR amplification employed V5-V6 region-specific primers for bacterial 16S rRNA: 799F (5′-AACMGGATTAGATACCCKG-3′) and 1115R (5′-AGGGTTGCGCTCGTTG-3′). After amplification, the products were detected via 2% agarose gel electrophoresis. The PCR products were purified using the AxyPrep DNA Gel Extraction Kit (Axygen, Union City, CA, USA) and quantified with the Quantus ™ Fluorometer (Promega, Madison, WI, USA). Library construction was performed using the NEXTFLEX^®^ Rapid DNA-Seq Kit (Bioo Scientific, Austin, TX, USA), and sequencing was conducted on the Illumina NovaSeq PE250 platform (Shanghai LingEn Biological Technology Co., Ltd., Shanghai, China).

### 2.3. Statistical and Bioinformatics Analysis

The raw sequencing data were quality-controlled using Trimmomatic software (version 0.30) [14]. Sequences were assembled using FLASH software (version 1.2.7) [15], and operational taxonomic units (OTUs) were clustered at 97% similarity using Usearch (version 10) [16]. To obtain taxonomic information for each OTU, the representative sequences of the 97% [17] similarity OTUs were classified using the Uclust algorithm (version 1.2.22q, confidence threshold 0.8), and the community composition of each sample was analyzed at different taxonomic levels. Alpha diversity indices (Observed species, Chao1, ACE, Shannon, and Simpson) were calculated using the “vegan” package in R (version 4.0.2). Principal coordinate analysis (PCoA) based on the Bray–Curtis distance, which was performed using the “vegan” package, and permutational multivariate analysis of variance (PERMANOVA) were conducted to assess the effect of sampling position on the microbial community [18]. Differentially abundant taxa were identified using LEfSe (linear discriminant analysis effect size) [19]. The functional annotation of bacterial communities was conducted with PICRUSt2 (version 2.5.2) [20].

### 2.4. Isolation and Purification of Seed-Endophytic Bacteria

Surface-disinfected seeds (0.5 g) were placed in a sterilized mortar, combined with 1.5 mL of sterile water, and homogenized by grinding using a sterile pestle. The resulting juice was diluted with sterile distilled water in a gradient from 10^−1^ to 10^−6^, and 200 μL of the 10^−4^, 10^−5^, and 10^−6^ dilutions was plated onto LB solid medium. The inoculated plates were placed in a 30 °C incubator and cultured in an inverted way for 3–5 days, with daily observations of colony growth. After colony formation, individual colonies were selected based on the morphological characteristics and repeatedly purified using the streak plate technique. The purified isolates were stored as culture suspensions in 30% (vol/vol) glycerol.

### 2.5. Evaluation of PGP Activity of Seed-Endophytic Bacteria

The ability of the strains to dissolve phosphate was qualitatively assessed using inorganic and organic phosphate media [21]. The activated strains were picked as single colonies using a sterile toothpick and inoculated onto the detection medium. The cultures were incubated at 30 °C for 5 days. A clear halo surrounding the colony indicated phosphate dissolution activity. The phosphate solubilization activity was determined by the ratio of the diameter of the clear zone (D) to the diameter of the colony (d) [22].

IAA production was quantified colorimetrically using Salkowski reagent in nutrient broth containing 0.1% tryptophan [23]. The siderophore-producing ability of the strains was assessed using CAS agar medium [24]. Activated strains were inoculated onto CAS assay plates by transferring single colonies with sterile toothpicks and incubated at 30 °C for 5 days. The formation of an orange halo zone around the colonies on blue CAS agar plates indicated siderophore production, and this ability was determined by the ratio of the halo diameter (D) to the colony diameter (d) [25].

The nitrogen-fixing capability was assessed using Ashby’s nitrogen-free medium [26]. The strains were inoculated into Ashby’s medium and incubated at 30 °C for 7 days. The ability of the strains to grow on the medium indicates their nitrogen-fixing capacity.

The activities of protease, cellulase, lipase, and amylolytic enzymes were determined on agar plates containing skim milk powder, sodium carboxymethyl cellulose, tributyrin, and starch, respectively [27,28]. Activated strains were inoculated onto four assay media by transferring single colonies with sterile toothpicks and incubated at 30 °C for 3 days. The enzyme-producing ability of the strains was quantified using the ratio of the hydrolysis zone diameter (D) to the colony diameter (d).

### 2.6. Seed Endophyte-Mediated Growth Promotion Assay in Potted Wheat

The activated strains were inoculated into LB medium at a 1% inoculum and cultured overnight at 30 °C, 180 rpm, until the OD600 reached 0.8. The bacteria were collected by centrifugation, and the pellet was resuspended in an equal volume of sterile water to obtain a bacterial suspension. Wheat seeds were washed with sterile water, disinfected with 75% alcohol for 15 s, immersed in 2% sodium hypochlorite for 10 min, and washed 5 times with sterile water. The disinfected wheat seeds were soaked in sterilized distilled water for 10 h. After soaking, the wheat seeds were placed in a constant-temperature incubator, in the dark, to induce germination. Germinated wheat seeds were sown in plastic pots sterilized with 75% ethanol, each filled with a commercial potting mix that had undergone a dual autoclaving procedure (121 °C, 15 psi/0.103 MPa, 30 min per cycle, with a 24 h interval between cycles). Prior to sowing, the sterility of the soil was confirmed by preparing soil suspensions, performing serial dilution (10^−1^ to 10^−6^) and plating on LB agar (for bacteria) and PDA (for fungi). After a 7-day incubation at 30 °C and 28 °C, respectively, no microbial growth was observed. After the seedlings developed two true leaves, each plant was inoculated via root drenching with either 5 mL of sterile water (negative control) or 5 mL of bacterial suspension (treatment group). The bacterial solution was applied every 7 days to ensure the stable presence of the bacterial community. To study the effect of the strain on wheat seedling growth, plants were harvested for growth and physiological measurements after 21 days of growth at room temperature (22 °C, 16 h light, 8 h dark). Superoxide dismutase (SOD), peroxidase (POD), malondialdehyde (MDA), and soluble protein were measured following the methods described in references [29,30,31], using assay kits from Suzhou Grace Biotechnology Co., Ltd. (Suzhou, China; product codes: G0102F, G0108F, G0110F, and G0417F, respectively). All measurements were conducted strictly according to the manufacturer’s instructions. Three replicates were used for each treatment.

### 2.7. Abiotic Stress Tolerance of L7 Strain

#### 2.7.1. Osmotic Stress Tolerance

In the osmotic stress tolerance test, liquid LB medium containing 10%, 20%, 30%, and 40% PEG6000 (Beijing Solarbio Science & Technology Co., Ltd., Beijing, China, Cat: P8250, CAS: 25322-68-3, MW 5400–6600 Da) was prepared, with medium without PEG6000 used as the control. The test strains were inoculated at a 1% inoculum, cultured at 30 °C, 180 rpm for 24 h, and the OD600 value was measured [32].

#### 2.7.2. Salt Tolerance

In the salt tolerance test, NaCl was added to LB medium at concentrations of 1%, 3%, 5%, 7%, 9%, and 11%, with the medium containing 1% NaCl used as the control. The test strains were inoculated at a 1% inoculum, cultured at 30 °C, 180 rpm for 24 h, and the OD600 value was measured [33].

#### 2.7.3. pH Tolerance

In the acid–base tolerance test, HCl or NaOH was added to the medium to adjust the pH to 5, 6, 7, 8, 9, 10, and 11, with the medium at pH 7 used as that for the control group. The test strains were inoculated into the liquid medium at a 1% inoculum, cultured at 30 °C, 180 rpm for 24 h, and the OD600 value was measured [33].

### 2.8. Identification of Strain L7

Morphological and biochemical characterization: The purified strain L7 was inoculated onto LB solid medium using the streak plate method and incubated in an inverted way at 30 °C until single colonies appeared. Physiological and biochemical tests, including catalase, methyl red, Voges–Proskauer (V-P), gelatin liquefaction, hydrogen sulfide production, and the sole carbon source utilization test, were performed on L7 in triplicate.

Molecular Biology Identification: Bacterial DNA was extracted from the colonies using a bacterial DNA extraction kit. The bacterial genomic DNA was amplified with universal primers 27F: 5′-AGAGTTTGATCCTGGCTCAG-3′ and 1492R: 5′-TACGGCTACCTTGTTACGACTT-3′. After PCR amplification, the products were detected by 1.0% agarose gel electrophoresis. The PCR products were sequenced by BGI (Shenzhen, China). The sequencing results were compared online with the NCBI gene database [34]. A phylogenetic tree was constructed using MEGAX software (version 10.2.6).

### 2.9. L7 Sample Preparation and Genomic Sequencing

The L7 strain was inoculated into LB liquid medium and cultured overnight at 30 °C, 180 rpm. Once the strain reached the logarithmic growth phase, the bacterial suspension was collected and centrifuged at 4 °C, 5000× *g* for 5 min. The supernatant was discarded, and the bacterial pellet was collected in a 1.5 mL centrifuge tube. The sample was then rapidly frozen in liquid nitrogen and stored at −80 °C.

The samples were sent to Novogene Bioinformatics Technology Co., Ltd. (Beijing, China), and sequencing was performed using the Illumina PE 150 sequencing platform. The experimental procedure followed the provided standard protocol, including sample quality assessment, library construction, library quality control, and library sequencing. The raw data were processed and filtered to obtain Clean Data. The Clean Data were assembled using SOAP denovo (version 2.04) [35,36], SPAdes (version 3.15.5) [37], and ABySS (version 2.3.8) [38] assembly software and finally integrated using CISA software (version 1.3) [39]. The preliminary assembly results were optimized and gap-filled using gapclose (version 1.12). The final assembly was obtained by filtering out fragments shorter than 500 bp, followed by evaluation, statistical analysis, and subsequent gene prediction.

### 2.10. Gene Composition Analysis and Functional Annotation

Gene composition analysis includes coding gene prediction, non-coding RNA (ncRNA) prediction, repeat sequence prediction, gene island prediction, phage prediction, and CRISPR prediction. In functional annotation, the protein sequences of the predicted genes were aligned with various functional databases (NR, COG, KEGG, GO, Pfam, SwissProt, CAZy, TCDB) using Diamond (evalue ≤1 × 10^−5^). For each sequence, the alignment with the highest score (default identity ≥40%, coverage ≥40%) was selected for annotation. Using the KEGG database, genes related to IAA production, phosphate solubilization, nitrogen fixation, siderophore production, osmotic stress tolerance, and salt tolerance were analyzed. To identify potential pathogenicity-related genes, the genome of *B. altitudinis* L7 was queried against the Pathogen–Host Interactions database (PHI-base, version 4.17) using BLASTp (version 2.13.0) with the following thresholds: evalue < 1 × 10^−5^, sequence identity > 30%, and alignment coverage > 70%.

### 2.11. Statistical Analysis

The data are presented as mean ± standard deviation (SD). One-way analysis of variance (ANOVA) was performed using IBM SPSS Statistics (version 26.0.0.0) software, followed by the LSD post hoc test at a significance level of *p* ≤ 0.05. All of the statistical analysis results were plotted using GraphPad Prism 8 (version 8.0.2) and R (version 4.0.2) software.

## 3. Results

### 3.1. Community Composition and Diversity Analysis

To investigate the community composition of endophytic bacteria in *S. pennata*, seeds were collected from two locations in the China Gurbantunggut Desert: the hinterland (site A) and the southern margin (site B). The V5-V6 hypervariable regions of the bacterial 16S rRNA gene were amplified and sequenced, resulting in 224,854 high-quality reads. The sequencing depth was sufficient to capture most of the microbial diversity in the samples (Figure S1).

Based on 97% sequence similarity, a total of 775 operational taxonomic units (OTUs) were identified, spanning 21 phyla, 38 classes, 97 orders, 169 families, 395 genera, and 82 species. Among these, 186 OTUs (24%) and 135 OTUs (17.4%) were unique to sites A and B, respectively, while 454 OTUs (58.6%) were shared between the two sites (Figure S2).

**Figure 1:**
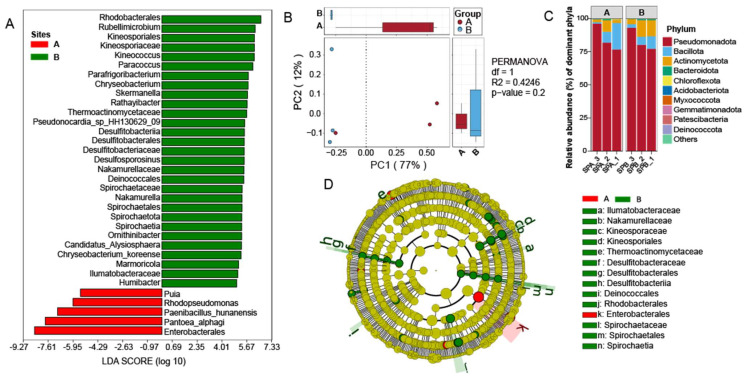
Analysis of endophytic community composition and diversity in *S. pennata* seeds. (**A**) Differential taxa identified between the two sites (*p* < 0.05, LDA > 2.0). (**B**) Principal coordinate analysis (PCoA) of endophytic bacterial communities from the two sampling sites. (**C**) Relative abundances of endophytic bacteria at the phylum level. (**D**) LEfSe evolutionary branch diagram illustrating significantly different endophytic bacterial taxa.

At the phylum level, Pseudomonadota, Bacillota, and Actinomycetota each showed relative abundances exceeding 1% (Figure 1C). Pseudomonadota was the dominant phylum, accounting for 84.21% of the total community. At site A, Bacillota and Actinomycetota represented 9.70% and 4.32%, respectively, whereas at site B, their proportions were 6.01% and 9.55%. At the genus level, 10 genera exhibited relative abundances above 1% (Figure S3A). Among these, *Burkholderia*, *Caballeronia*, *Paraburkholderia*, *Candidatus Liberibacter*, *Dietzia*, and *Massilia* were common to both sites with relative abundances above 1%. *Exiguobacterium*, *Paenibacillus*, *Pantoea*, and *Pseudomonas* exceeded 1% only at site A, while *Acinetobacter* exceeded 1% only at site B. Notably, *Candidatus Liberibacter* was the dominant genus, comprising 12.17% and 45.24% of the community at sites A and B, respectively. In addition, *Bacillus*, *Peribacillus*, and *Microbacterium* were detected at both sites, although with relatively low abundances. *Bacillus* accounted for 0.20% at site A and 0.58% at site B; *Peribacillus* was present at 0.0019% at both sites; and *Microbacterium* represented 0.018% and 0.088% at sites A and B, respectively (Figure S3B).

The endophytic bacterial communities in the seeds showed no significant differences in the overall diversity between the two sites. Although site B consistently showed higher alpha diversity values across Chao1, Observed_species, ACE, Shannon, and Simpson indices, these differences were not statistically significant (Table S1), indicating comparable species richness and diversity. For beta diversity, the analysis of NMDS (Non-metric Multidimensional Scaling) revealed differences in the composition of microbial communities between the two sites (Figure S4), and principal coordinate analysis (PCoA) revealed partial separation of the communities along the PC1 axis (Figure 1B). However, PERMANOVA analysis did not support a significant difference between the sites (*p* = 0.2, R^2^ = 0.4246). Despite the overall similarity in diversity, LEfSe analysis (*p* < 0.05, LDA > 2.0) identified 34 taxa with significantly different relative abundances between the two sites (Figure 1A). Notably, site B featured a broader phylogenetic distribution and slightly higher biomarker diversity (Figure 1D).

Based on the PICRUSt2 predictions of KEGG metabolic functions, the endophytic microbiota in *S. pennata* seeds predominantly participate in amino acid metabolism (including cysteine, methionine, arginine, proline, valine, leucine, and isoleucine), carbon metabolism (such as glycolysis/gluconeogenesis, pentose phosphate pathway, pyruvate metabolism, propanoate metabolism, and glyoxylate and dicarboxylate metabolism), fatty acid degradation, carbon fixation, nitrogen metabolism, and methane cycling. In addition, key molecular mechanisms like bacterial secretion systems, ABC transporters, two-component systems, and porphyrin and chlorophyll metabolism were also enriched (Figure S5). This enrichment reflects the metabolic specialization of certain genera within the community. Notably, *Bacillus* may promote host stress response signaling by secreting plant growth-promoting substances, such as auxin-like compounds, which could drive the enrichment of these pathways. Therefore, isolating and characterizing the metabolic capabilities of such functional genera are essential for understanding microbial adaptation to environmental stress.

### 3.2. Isolation of Endophytic Bacteria from S. pennata Seeds

Nine endophytic bacterial strains were isolated from *S. pennata* seeds. Based on the 16S rRNA sequence alignment, these strains were classified into three genera: *Bacillus* (six strains: L1, L3, L7, L9, L10, and L11), *Peribacillus* (two strains: G5 and G8), and *Microbacterium* (one strain: G10) (Table S2).

Further functional characterization revealed that all strains except G10 produced indole-3-acetic acid (IAA) at concentrations ranging from 3.57 to 19.99 mg/L (Table S3, Figure S6A,B). Strains L7, L9, L10, and L11 solubilized both organic phosphate (solubilization index: 1.31–2.26) and inorganic phosphate (1.91–2.20) (Table S3, Figure S6C,F). All strains demonstrated siderophore synthesis capacity with production indices of 2.88–4.23 (Table S3, Figure S6E). Nitrogen fixation capability was confirmed in L1, L7, L9, L10, and L11 strains (Table S3, Figure S6D).

Protease production was observed in all strains except G10, with hydrolysis-zone-to-colony-diameter ratios (D/d) ranging from 1.39 to 3.35 (Table S4, Figure S7A). Additionally, cellulase activity was detected in L1, L3, L7, L9, L10, and L11 (D/d: 3.54–6.83) (Table S4, Figure S7B), amylase activity was observed in L1, L3, L9, L10, G5, and G8 (D/d: 1.18–2.84) (Table S4, Figure S7C), and lipase activity was present universally across all strains (D/d: 1.61–3.40) (Table S4, Figure S7D).

**Figure 2:**
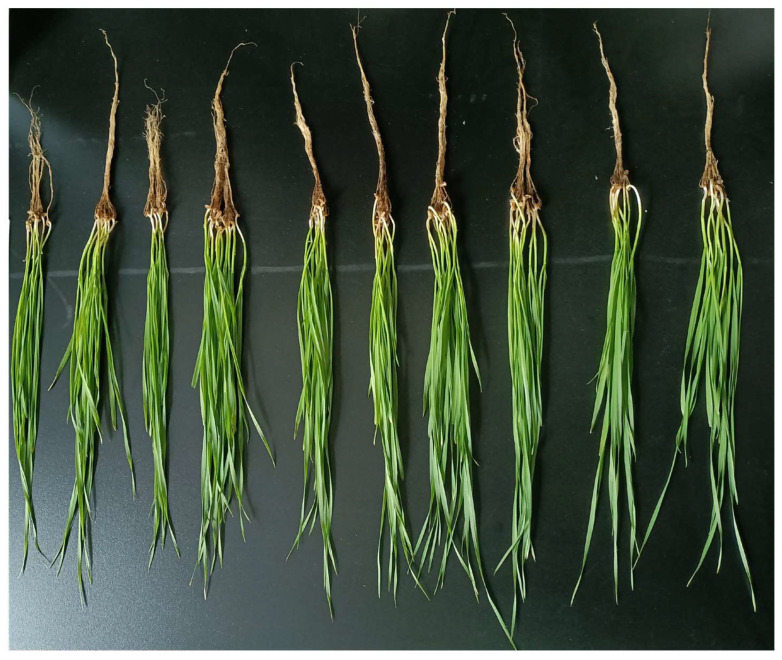
Growth of wheat seedlings under different bacterial treatments. From left to right: control (CK) and treatments with bacterial strains L1, L3, L7, L9, L10, L11, G5, G8, and G10.

**Figure 3:**
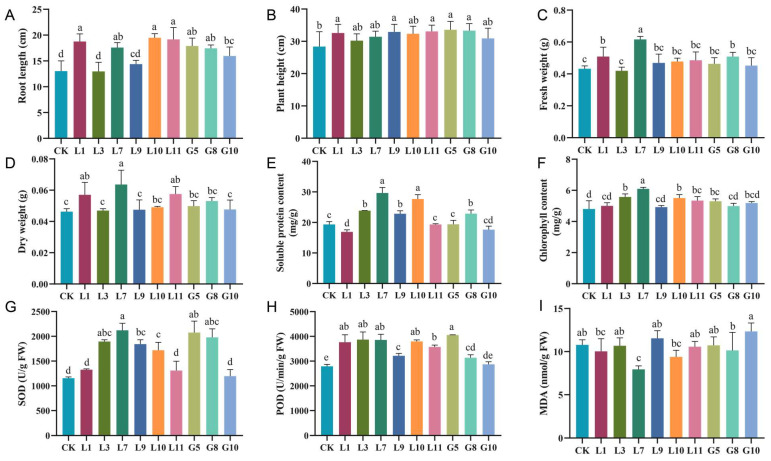
Growth-promoting effects of bacterial strains on wheat seedlings. (**A**) Root length. (**B**) Plant height. (**C**) Fresh weight. (**D**) Dry weight. (**E**) Soluble protein content. (**F**) Chlorophyll content. (**G**) Superoxide dismutase (SOD) activity. (**H**) Peroxidase (POD) activity. (**I**) Malondialdehyde (MDA) content. Differences among treatments were assessed by one-way ANOVA. Different lowercase letters indicate statistically significant differences (*p* < 0.05). Error bars represent mean ± standard deviation (SD).

The isolated bacterial strains promoted wheat seedling growth to varying degrees (Figure 2). Specifically, root length increased by 10.36% to 49.49% (Figure 3A), plant height by 6.40% to 18.32% (Figure 3B), fresh weight by 4.65% to 42.58% (Figure 3C), and dry weight by 1.46% to 37.49% (Figure 3D). The soluble protein content rose by 0.09% to 53.36% (Figure 3E), and the chlorophyll content increased between 2.48% and 26.85% (Figure 3F).

Moreover, the bacteria significantly enhanced the antioxidant enzyme activities in wheat leaves: the SOD activity increased by 3.67% to 83.85% (Figure 3G), and the POD activity increased by 2.75% to 45.25% (Figure 3H). Notably, strain L7 reduced the MDA content by 26.37% (Figure 3I), indicating its strong potential in alleviating oxidative stress. Overall, strain L7 exhibited the most pronounced growth-promoting effects across all of the measured parameters.

### 3.3. Genomic Characteristics of Bacillus altitudinis Strain L7

Strain L7 possesses the ability to solubilize both organic and inorganic phosphorus, produce indole-3-acetic acid (IAA) and siderophores, fix nitrogen, and secrete various enzymes including protease, lipase, and cellulase. Additionally, it exhibits tolerance to salt and osmotic stress (Figure S8). Morphological, physiological and biochemical, and molecular analyses identified strain L7 as *Bacillus altitudinis* (Figure S9, Table S5). To further elucidate its functional mechanisms, genomic sequencing and annotation were conducted on strain L7.

**Figure 4:**
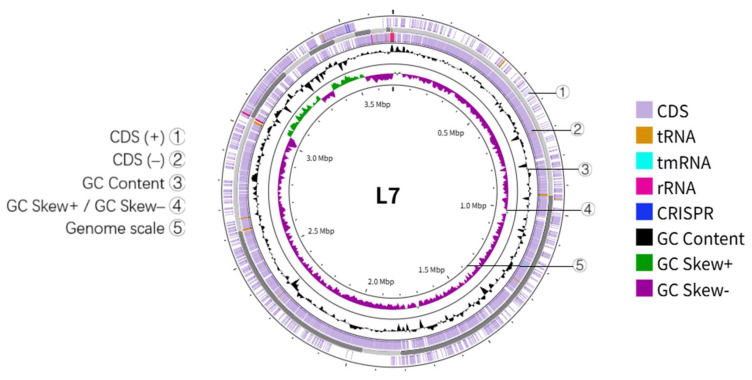
The genomic map of L7. From the inside out: genomic coordinates, GC skew (green indicates G > C, purple indicates G < C), GC content, and genes on the positive and negative strands.

The genome of strain L7 is 3,682,572 bp in length with a GC content of 41.73%. Annotation identified 3880 protein-coding genes, 71 tRNAs, 83 ncRNAs, 8 5S rRNAs, 1 16S rRNA, 1 23S rRNA, 2 sRNA genes, 133 LTRs, 44 DNA repeat elements, 46 LINEs, 8 SINEs, 2 prophages, and 4 genomic islands (Figure 4, Table S6).

**Figure 5:**
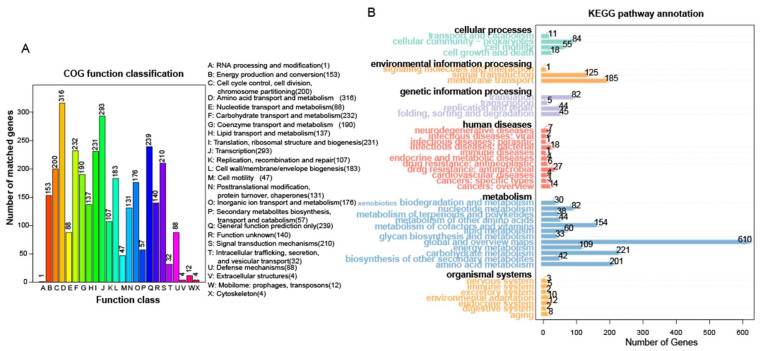
Gene functions annotated in the COG (**A**) and KEGG (**B**) databases.

Based on the COG and KEGG annotations, 2891 and 3678 protein-coding genes were assigned functions, respectively (Figure 5, Table S6). In both databases, the largest proportions of genes were related to amino acid transport and metabolism (accounting for 10.93% in COG and 5.46% in KEGG) and carbohydrate metabolism (8.02% in COG and 6% in KEGG), indicating the dominance of the genes involved in these pathways. These findings align with the functional predictions from PICRUSt2 for the bacterial community. PHI-base analysis revealed that strain L7 contains only the *cel-EIIB* gene and lacks key pathogenicity-related genes—including *celZ*, *celY*, *egl*, *eglXoB*, *pel*, *peh*, *pnl*, and *amy*—which are commonly associated with plant pathogenicity (see Supplementary Excel S1). These findings suggest a low risk of plant pathogenic potential for strain L7.

**Figure 6:**
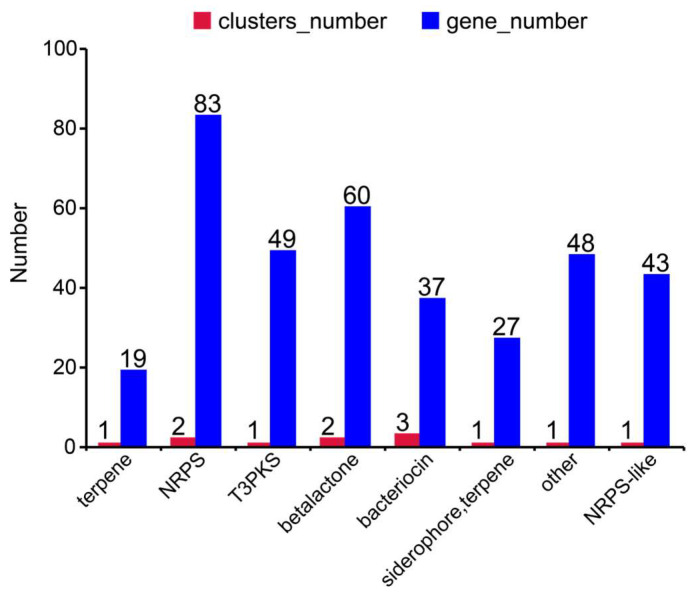
Distribution of secondary metabolite biosynthetic gene clusters and gene counts.

Strain L7 harbors seven gene clusters involved in secondary metabolite biosynthesis (Figure 6), classified as follows: terpene, NRPS, T3PKS, betalactone, bacteriocin, siderophore, terpene, and NRPS-like. Among these, bacteriocin gene clusters were most abundant (three), followed by NRPS and betalactone (two each); all other types contained a single cluster. Notably, the NRPS gene cluster comprised the highest number of genes (83). These results indicate that strain L7 possesses the capacity to synthesize antimicrobial peptides, complex peptide compounds, terpenes, polyketides, siderophores, and enzyme inhibitors. This functional versatility suggests enhanced environmental adaptability and ecological competitiveness, enabling the strain to execute antimicrobial defense, resource competition, and survival stress responses through diversified secondary metabolites.

In the tryptophan metabolic pathway, the genome of strain L7 contains indole-3-acetamide hydrolase (EC 3.5.1.4) and acetaldehyde dehydrogenase (EC 1.2.1.3) (Figure S10) but lacked tryptophan-2-monooxygenase (EC 1.13.12.3), indicating an incomplete IAA synthesis pathway. Additionally, the tryptophan biosynthesis pathway genes *trpABCDEF* were identified (Figure S11, Table S7), which were consistent with the in vitro IAA production results of the strain.

Regarding nitrogen fixation, genes related to this process, such as *nifS* and *nifU*, were detected in the L7 genome (Table S7); however, a complete nitrogenase gene cluster was not identified. Additionally, genes encoding glutamate dehydrogenase (*gudB*, *rocG*) and nitrate/nitrite reductase (*nasA*, *nirB*, *nirD*) involved in nitrogen metabolism were present, enabling the reduction of L-glutamate and nitrate to ammonia (Figure S12).

The siderophore gene cluster in strain L7 includes biosynthetic genes such as *dhbBEF*, *vibBE*, *entABCE*, as well as transport and secretion genes, including *mxcEF* and *fhuB*/*C*/*D*/*G* (Table S7). Three complete siderophore synthesis pathways were identified in the L7 genome (Figure S13).

Genes related to phosphate solubilization identified in strain L7 include acid and alkaline phosphatases (*appA*, *phoA*), phosphate transporters (*pstSCAB*, *pit*), the carbon–phosphorus lyase operon (*phnAB*), and regulatory genes such as *phoB* and *phoE* (Figure S14, Table S7). These genes likely contribute to the strain’s observed phosphate-solubilizing ability in vitro.

Genes related to salt tolerance and osmotic stress tolerance identified in strain L7 include those encoding the Na^+^ efflux system genes (*mrpABCDEFG*/*mnhABCDEFG*, *nhaC*), K^+^ uptake genes (*trkA*), ectoine genes (*ectB*), glycine betaine transporter genes (*betL*), glycine betaine/proline transport system genes (*proVWX*, *opuABCD*), and proline metabolism (*proABC*). Additionally, molecular chaperone genes (*dnaJK*, *groES*, *groEL*, *hsp20*) were detected, which may aid in adaptation to salt and osmotic stress (Table S7).

## 4. Discussion

This study elucidated the endophytic bacterial community structure and plant growth-promoting (PGP) functional traits in *S. pennata* seeds, showing similar diversity across sampling sites but distinct functional genes and taxonomic profiles. Among nine isolated strains, L7 stood out with exceptional PGP and stress tolerance capabilities. Its genome contains abundant genes related to IAA synthesis, nitrogen fixation, phosphate solubilization, siderophore production, and salt and osmotic stress tolerance, underpinning its adaptation to extreme environments and highlighting its potential as an effective PGP inoculant.

The species richness and diversity of endophytic bacteria in *S. pennata* seeds from two sampling sites (approximately 100 km apart) did not differ significantly. The alpha diversity indices are key ecological parameters used to analyze community composition and biodiversity distribution characteristics [40]. Among them, the Observed species, Chao1, and ACE indices can be used to measure the species richness of seed-endophytic bacteria, while the Shannon and Simpson indices can be used to assess bacterial diversity [41]. Higher α-diversity indicates a more complex ecosystem that can better withstand environmental disturbances [42]. These results indicated that species richness and diversity at site B were higher than that at site A, potentially due to differences in host genotype, desert environmental factors (e.g., temperature, humidity, soil properties), as well as seed maturity and storage conditions. NMDS and beta diversity analyses revealed some trends of differentiation between bacterial communities from the two sites; however, these inter-group differences were not statistically significant (*p* > 0.05), suggesting that host factors may limit microbiota differentiation across sites.

At the phylum level, Pseudomonadota, Bacillota, and Actinomycetota formed the core endophytic microbiota shared between the two sampling sites, consistent with reports of these phyla dominating seed-associated microbiomes in other plant species [43,44]. Pseudomonadota was the most abundant phylum (84.21%), aligning with the findings in *Panax notoginseng* [45], *Picea abies* [46], and *Cannabis sativa* [47]. Culturable bacteria previously isolated from site A seeds (*S. pennata*) belonged to Pseudomonadota and Bacillota, whereas strains isolated from site B in this study are affiliated with Bacillota and Actinomycetota, consistent with the high-throughput sequencing results. At the genus level, *Candidatus Liberibacter* emerged as the dominant shared genus; its obligate phloem-associated lifestyle may be linked to seed physiological regulation or adaptation to environmental stress. Notably, previously isolated *Pantoea* and *Paenibacillus* from site A, as well as *Bacillus*, *Peribacillus*, and *Microbacterium* from site B, were successfully detected via cultivation-independent methods. However, *Candidatus Liberibacter* was only identified via sequencing, highlighting the limitations of traditional cultivation. In summary, the synergistic application of uncultured and traditional cultivation methods can overcome the limitations of single techniques, providing more comprehensive microbial resources and a theoretical basis for understanding plant–microbe interactions and developing desert ecological restoration strategies.

Endophytic bacteria are widely recognized for their role in promoting plant growth. They help maintain soil fertility, reduce the need for chemical fertilizers, and enhance crop productivity [48]. Pot experiments demonstrated that inoculation with isolated bacterial strains significantly improved host plant growth. For instance, treatment with strain G5 increased plant height by 18.32%, while L10 enhanced root length by 49.49%. Notably, strain L7 led to substantial increases in fresh weight (42.58%), dry weight (37.49%), chlorophyll content (26.85%), and soluble protein content (53.36%). In this study, strain L7 further increased the soluble protein content and fresh weight in plant leaves compared to other documented strains [49]. In addition to growth promotion, the inoculated strains also elevated antioxidant enzyme activities: SOD activity increased by 3.67%–83.85%, and POD activity increased by 2.75%–45.25%. Furthermore, L7 significantly reduced the MDA content by 26.37%, indicating its potential to mitigate oxidative damage through activation of the plant’s antioxidant defense system. Among them, the increase in SOD was comparable to that of *Crocus sativus* bacteria, but the increase in POD and the degree of MDA inhibition were lower, which may reflect the specificity of the strain–host interaction [50]. Overall, L7 demonstrated the most pronounced plant growth-promoting effects among the tested strains.

The L7 genome contains several genes associated with phosphate acquisition and regulation, including phosphate transporter genes (*pstSCAB*, *pit*), the *phoBERHL* regulatory cluster, and phosphatase genes (*appA*, *phoA*). Under phosphate-replete conditions, the pst system is proposed to form an inhibitory complex with *phoR*, thereby preventing *phoB* activation. Phosphate restriction triggers the transition of *phoR* to its active state, wherein autophosphorylated *phoR* transfers its phosphoryl group to *phoB*. Restoration of the repressed state occurs upon transition to phosphate-sufficient environments [51]. Additionally, the two-component system (*phoB*-*phoR*) under low-phosphate conditions involves the autophosphorylation of *phoR* (histidine kinase), which activates *phoB* (response regulator, *phoB*-P); *phoB*-P directly binds promoters of target genes (e.g., *phoA*, *phoE*, *appA*) [52,53]. Notably, the alkaline phosphatase *phoA* is capable of hydrolyzing a range of organic phosphorus compounds (including monoesters, diesters, triesters, and sulfate esters) to release inorganic phosphate (Pi), enabling efficient phosphorus utilization [54]. Consistent with this, in vitro assays confirmed that L7 is capable of solubilizing both inorganic and organic phosphate. Strain L7 exhibited broader genetic potential, harboring *appA*, *phoBERHL*, and *phnAB* beyond the core components, compared to other documented strains [55].

In the tryptophan biosynthesis pathway of the L7 genome, a complete set of *trpABCDEF* genes was identified. This is consistent with previous findings, such as the presence of *trpABD* in the genome of *Sphingomonas* sp. LK11 [56] and *trpABFCDE* in strain Q2H2 [33], both of which are involved in IAA-related tryptophan biosynthesis.

L7 demonstrated nitrogen-fixing capacity through growth and halo formation on Ashby nitrogen-free medium. Key genes for iron–sulfur cluster biosynthesis (*nifS*, *nifU*, *iscU*, and *sufBCD*) were identified in the L7 genome. According to the established literature [57], the *isc*/*suf*/*nif* systems constitute the primary pathway for iron–sulfur (Fe-S) cluster assembly in diazotrophic bacteria, thereby providing theoretical support for the identification of nitrogen fixation genes in strain L7. Two ammonia-generating pathways were found in the nitrogen metabolism pathway of L7: one catalyzed by glutamate dehydrogenases encoded by *gudB* and *rocG*, which convert L-glutamate into ammonia; the other involving nitrate and nitrite reductases encoded by *nasA*, *nirB*, and *nirD*, which sequentially reduce nitrate to nitrite and then to ammonia. The ammonia metabolic pathway in strain L7 demonstrates the evolutionary conservation of ammonia production strategies in diazotrophic bacteria, consistent with the patterns documented in other reported strains [33]. Additionally, nitrogen metabolism-related genes (*glnA*, *narK*, *nasA*, *nrtP*, *nos*, and *gltBCD*) were identified in the L7 genome. This genetic profile shares conserved genes *nifU*, *glnA*, and *gltBD* with the nitrogen-fixing sugarcane endophyte *Enterobacter roggenkampii* ED5 [58]. Notably, L7 uniquely encodes a nitrate transport system (*narK*, *nrtP*) and nitrous oxide reductase (*nos*), demonstrating that strain L7 possesses sophisticated nitrogen cycling capabilities.

Iron mostly exists in the form of poorly soluble oxides, and the amount of bioavailable soluble iron is relatively low. However, some microorganisms can produce siderophores that bind to ferric iron (Fe^3+^), converting the plant-unavailable iron into ferrous iron (Fe^2+^), which can be readily absorbed and utilized by plants, thereby enhancing plant iron uptake and utilization efficiency [59]. The whole genome of *Klebsiella* sp. D5A reported in the literature only harbors the siderophore synthesis genes *entABCDEF* [60]. However, in this study, the genome of strain L7 was found to contain gene clusters encoding the ferrichrome and other Fe^3+^-siderophore ABC transporters (*fhuBCDG*) as well as core gene clusters *entABCE*, *dhbBEF*, *vibBE*, and *mxcEF* responsible for the biosynthesis of siderophores, such as vibriobactin, bacillibactin, and enterobactin, enabling efficient iron chelation and uptake. This indicates that strain L7 employs a dual iron acquisition strategy, capable of both synthesizing high-affinity siderophores autonomously and utilizing exogenous siderophores (e.g., hydroxamate complexes), conferring a significant competitive advantage under iron-limited conditions. Multiple functional genes related to siderophores were identified in the L7 genome, and combined with the siderophore assay results, this demonstrates that strain L7 possesses the ability to secrete siderophores.

The L7 genome encodes genes for ectoine synthesis (*ectB*), glycine betaine transporter (*betL*), glycine betaine/proline transport systems (*proVWX*, *opuABCD*), and proline-related genes (*proABC*); these genes form a multilayered osmotic and salt–alkali resistance network associated with microbial adaptation to extreme environments [61]. The genes involved in osmotic sensing and regulation were also discovered in the L7 genome. The *mrpABCDEFG*/*mnhABCDEFG* and *nhaC* genes primarily alleviate salt–alkali stress via Na^+^/H^+^ antiport, while *trkA* balances osmotic pressure through K^+^ uptake [62,63]; these three systems synergistically maintain bacterial ion and pH homeostasis. In vitro experiments confirmed L7 tolerance to 9% NaCl and 40% PEG6000. Key genes for glycine betaine synthesis (*betA* and *betB*), a potassium transport system for K^+^ accumulation, and Na^+^/H^+^ antiporters (*nha*) for H^+^ import and Na^+^ export were identified in *Klebsiella* sp. D5A [60]. Notably, L7 additionally harbors the ectoine synthase gene *ectB*, *proABCVWX*, and *opuABCD*, enhancing the functional diversity of salt–alkali adaptation mechanisms.

The functional traits and plant-beneficial effects of *B. altitudinis* L7 support its classification as a microbial plant biostimulant. Microbial biostimulants are defined as microorganisms that enhance plant growth, nutrient-use efficiency, and abiotic stress tolerance through physiological modulation rather than direct nutrient supply [64]. L7 fits this definition: it produces indole-3-acetic acid (IAA), promoting root development and biomass accumulation; facilitates nitrogen fixation, phosphate solubilization, and siderophore production to improve nutrient availability and uptake; and enhances chlorophyll content and nutrient efficiency, consistent with previous findings [65,66]. Furthermore, L7 enhances plant stress tolerance by upregulating antioxidant enzymes (SOD, POD), increasing soluble proteins, and reducing malondialdehyde (MDA) levels [67]. Genomic analysis corroborates these functions, revealing genes related to IAA biosynthesis, nitrogen metabolism, phosphate transport, siderophore production, and osmoprotectant synthesis (e.g., ectoine). Collectively, these features go beyond conventional nutrient supplementation and position L7 as a promising microbial plant biostimulant.

The *B. altitudinis* L7 strain used in this study demonstrated strong potential for plant growth promotion. However, given previous reports of plant pathogenicity associated with certain *B. altitudinis* strains [68,69], we conducted a preliminary biosafety assessment. Genome comparison revealed that L7 carries only the *cel-EIIB* gene and lacks well-known pathogenicity-related genes, including *celZ*, *celY*, *egl*, and *eglXoB* [70,71]. Moreover, no key pectinase genes (*pel*, *peh*, *pnl*) [72] or amylase genes (*amy*) [73]—which are often implicated in virulence—were detected, suggesting a low-risk genetic profile. Consistent with this, wheat seedlings inoculated with L7 showed no visible disease symptoms, such as soft rot, wilting, or necrosis, further supporting its safety in this system.

Nonetheless, we acknowledge certain limitations. The absence of known pathogenic genes does not fully rule out potential risk, particularly in different host plants or environmental contexts. To address this, in vitro enzymatic assays to assess pectinase activity, as well as pathogenicity tests on apple and pear fruits, are planned as important future work. These studies will help provide a more comprehensive biosafety evaluation of strain L7.

## 5. Conclusions

Based on an integrated analysis of community structure and functional traits, this study uncovered the diversity and plant growth-promoting (PGP) potential of endophytic bacteria in *S. pennata* seeds from desert environments. High-throughput sequencing revealed that although endophytic bacterial communities exhibited comparable alpha diversity across sampling sites, they differed in taxonomic composition and predicted functional genes. Further isolation and systematic functional characterization of nine endophytic strains demonstrated their capacities for IAA production, nitrogen fixation, phosphate solubilization, siderophore synthesis, and diverse enzymatic activities. Among these, strain L7 exhibited superior PGP potential and notable tolerance to salt and osmotic stress. The genomic analysis of L7 revealed a suite of gene clusters associated with its functional capabilities, including phosphate transporter genes, nitrogen metabolism-related genes, and biosynthetic pathways for IAA and siderophores. In addition, genes linked to abiotic stress tolerance—such as those involved in Na^+^ efflux, K^+^ uptake, and compatible solute biosynthesis—were broadly distributed in the genome. The presence of these genes supports L7′s multifunctionality and adaptation to harsh desert conditions.

In conclusion, this study systematically explored the endophytic bacterial community of *S. pennata* seeds and identified strain L7 as a promising PGP candidate. By integrating cultivation-based assays and genomic analysis, the mechanisms underlying the plant growth-promoting (PGP) ability and stress resilience of L7 were elucidated. These findings provide valuable microbial resources and functional gene targets for desert ecosystem restoration and the development of sustainable, microbe-assisted green agriculture.

## Data Availability

The original data presented in this study are included in the article/supplementary material. Further inquiries can be directed to the corresponding authors.

## Supplementary Materials

The following supporting information can be downloaded at: https://www.mdpi.com/article/10.3390/microorganisms13081754/s1, Figure S1. Shannon curve diagram. The x-axis denotes sequencing depth, and the y-axis indicates the Shannon index. Figure S2. Distribution of endophytic bacteria in *Stipagrostis pennata* seeds between two sampling sites. Figure S3. Relative abundance of bacterial genera in *Stipagrostis pennata* seeds. (A) Bar plot of relative abundance of bacterial genera. (B) Heatmap of relative abundance of bacterial genera. Figure S4. NMDS analysis of endophytic bacterial samples from *Stipagrostis pennata* seeds. Figure S5. KEGG level 3 metabolic pathways of the endophytic bacterial microbiome in *Stipagrostis pennata* seeds. Figure S6. Determination of plant-growth-promoting abilities of bacterial strains. (A) Qualitative analysis of IAA production; from left to right: negative control, positive control, L1, L3, L7, L9, L10, L11, G5, G8, and G10. (B) Quantitative analysis of IAA production (different letters indicate significant differences among treatments by one-way ANOVA, *p* < 0.05). (C) Organic phosphate solubilization capacity. (D) Nitrogen fixation ability. (E) Siderophore production capacity. (F) Inorganic phosphate solubilization capacity. Figure S7. Enzyme production capacity of bacterial strains. (A) Protease production. (B) Cellulase production. (C) Amylase production. (D) Lipase production. Figure S8. Growth of strain L7 under abiotic stresses of varying PEG6000 (10–40%), pH (5–11), and salt concentration (1–11%). Figure S9. Morphological characteristics and phylogenetic analysis of strain L7. (A) Colony morphology of strain L7 on LB medium. (B) Phylogenetic tree of L7 constructed based on 16S rRNA gene sequences. Figure S10. KEGG pathway of tryptophan metabolism in strain L7 (enzymes present in strain L7 are highlighted in red). Figure S11. KEGG pathway of tryptophan biosynthesis in strain L7 (enzymes present in strain L7 are highlighted in red). Figure S12. KEGG pathway associated with nitrogen metabolism in strain L7 (enzymes present in strain L7 are highlighted in red). Figure S13. KEGG pathway associated with siderophore biosynthesis in strain L7 (enzymes present in strain L7 are highlighted in red). Figure S14. KEGG pathway of phosphate metabolism in strain L7 (enzymes present in strain L7 are highlighted in red). Table S1. Summary statistics of alpha diversity indices for endophytic bacteria in *Stipagrostis pennata* seeds. Table S2. Genus-level identification of endophytic bacteria based on 16S rRNA gene sequencing. Table S3. Evaluation of plant-growth-promoting (PGP) functions of endophytic bacteria in *Stipagrostis pennata* seeds. Table S4. Hydrolytic enzyme production capacity of endophytic bacteria in *Stipagrostis pennata* seeds. Table S5. Physiological and biochemical characteristics of strain L7. Table S6. Genomic features of endophytic strain L7. Table S7. Predicted genes associated with PGP and stress resistance functions in the genome of strain L7.
