# Supplementary material for: Community Structure, Growth-Promoting Potential, and Genomic Analysis of Seed-Endophytic Bacteria in Stipagrostis pennata
Source: Microorganisms. 2025 Jul 27;13(8):1754. doi: 10.3390/microorganisms13081754 (PMC12388186; doi:10.3390/microorganisms13081754)
Supplement: Supplementary file 1 [file microorganisms-13-01754-s001.zip › Supplementary figures and tables.pdf]

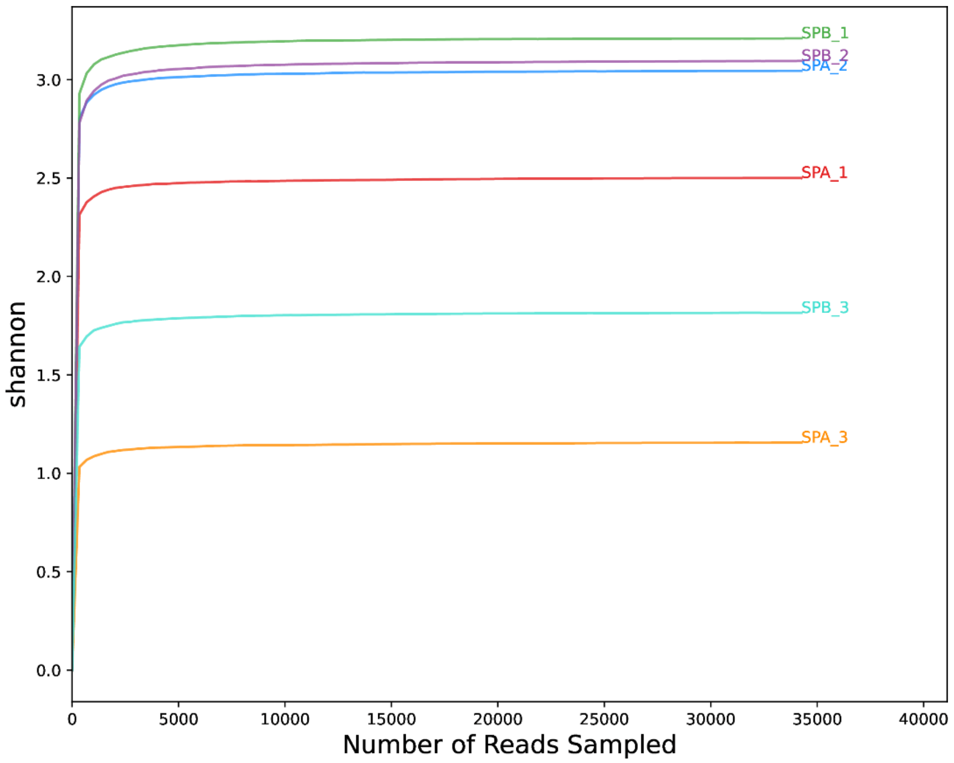

Figure S1. Shannon curve diagram. The x-axis denotes sequencing depth, and the y-axis indicates the Shannon index.

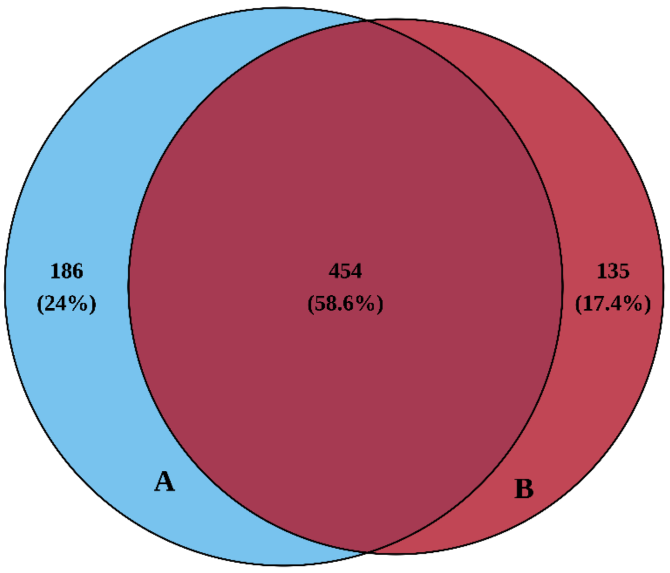

Figure S2. Distribution of endophytic bacteria in *Stipagrostis pennata* seeds between two sampling sites.

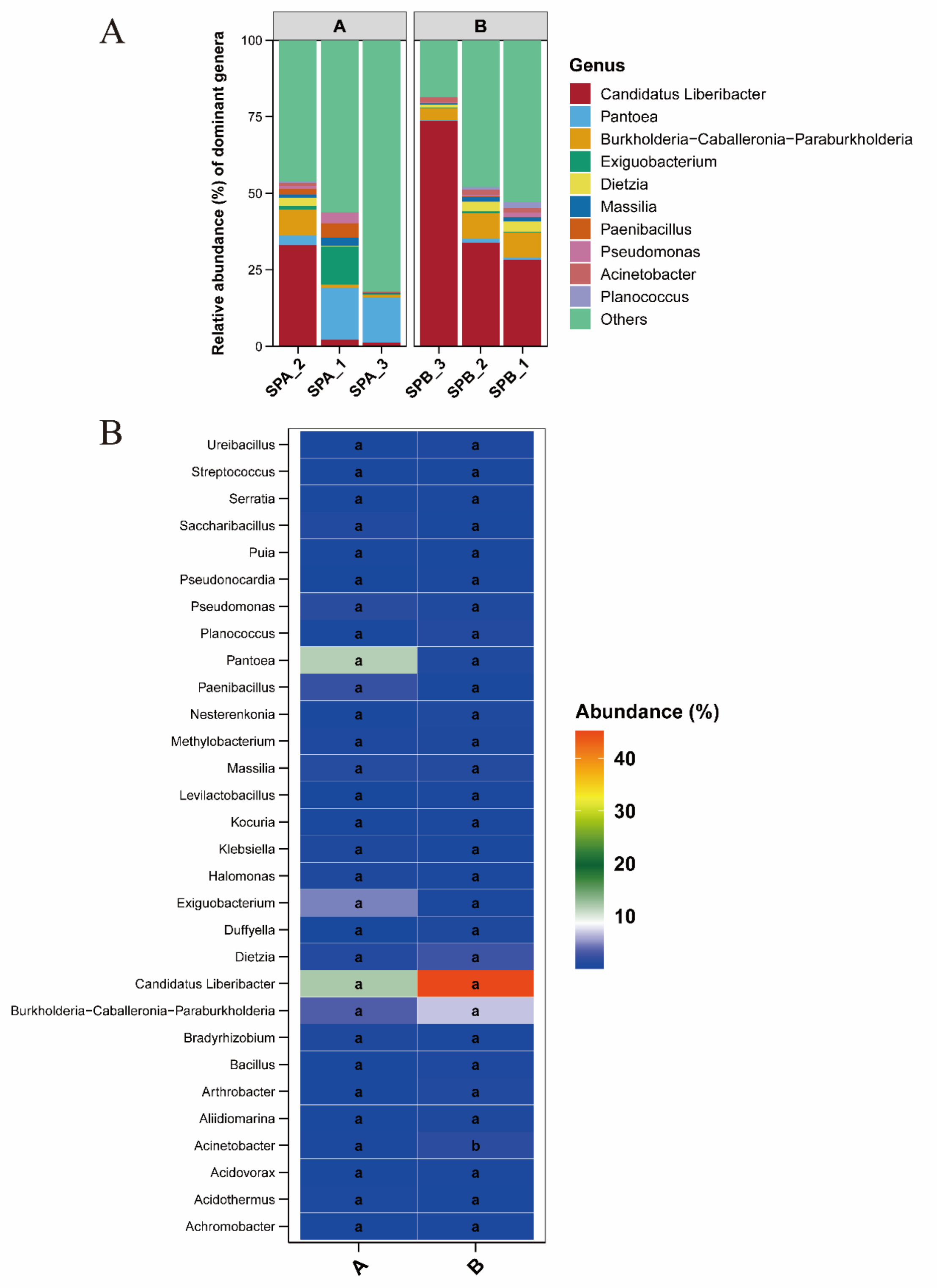

Figure S3. Relative abundance of bacterial genera in *Stipagrostis pennata* seeds. (A) Bar plot of relative abundance of bacterial genera. (B) Heatmap of relative abundance of bacterial genera.

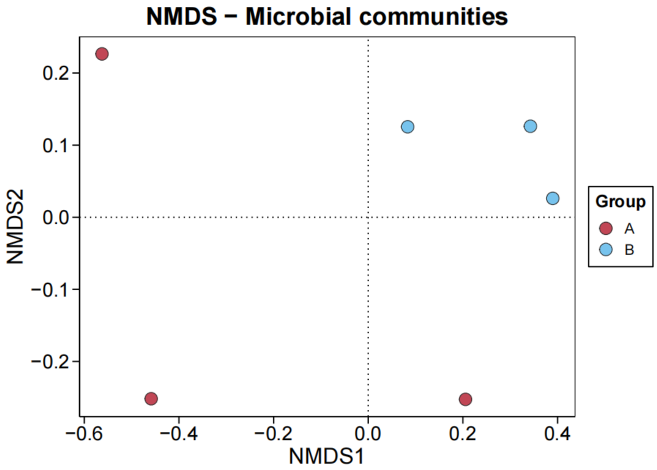

Figure S4. NMDS analysis of endophytic bacterial samples from *Stipagrostis pennata* seeds.

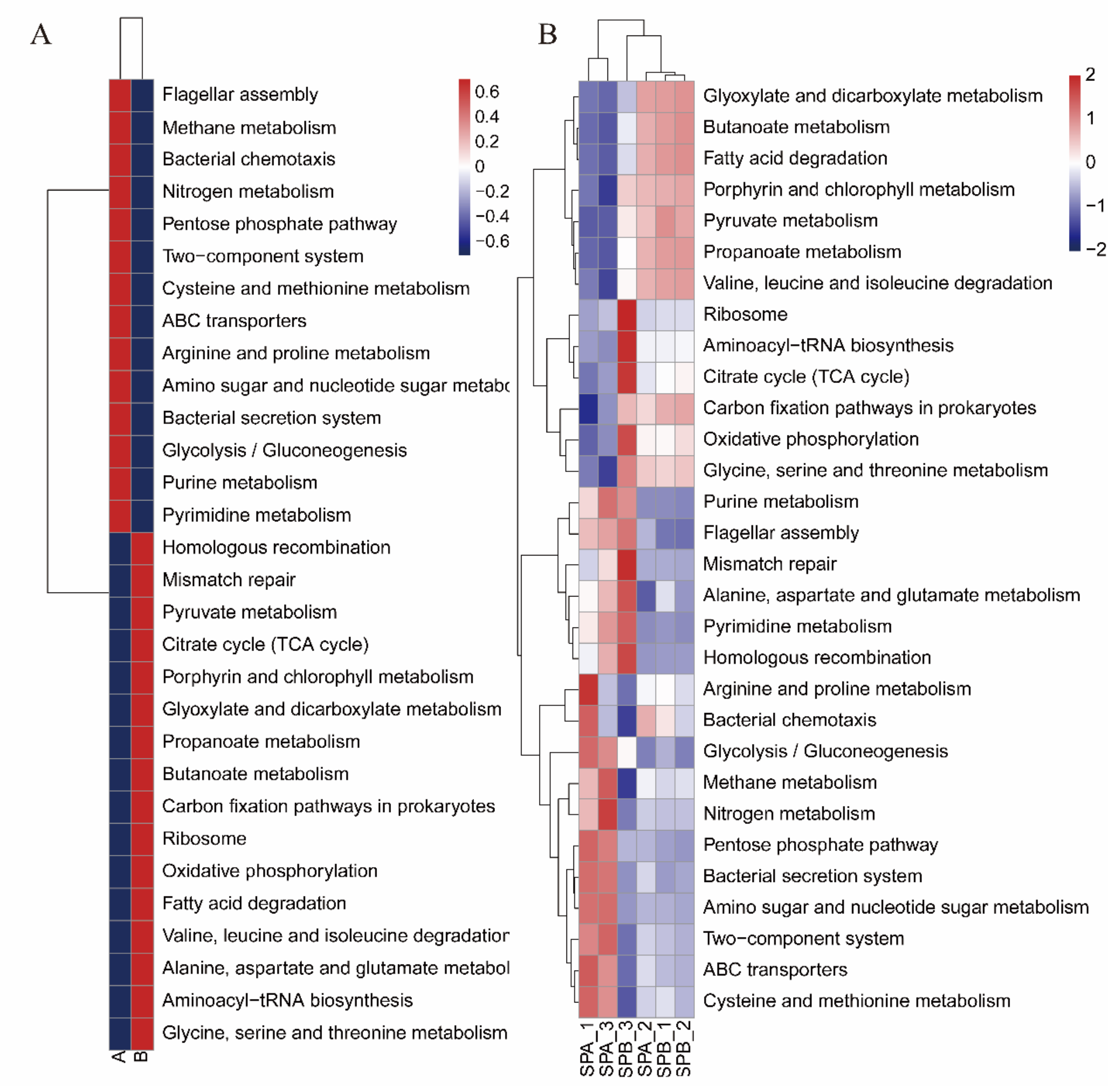

Figure S5. KEGG level 3 metabolic pathways of the endophytic bacterial microbiome in *Stipagrostis pennata* seeds.

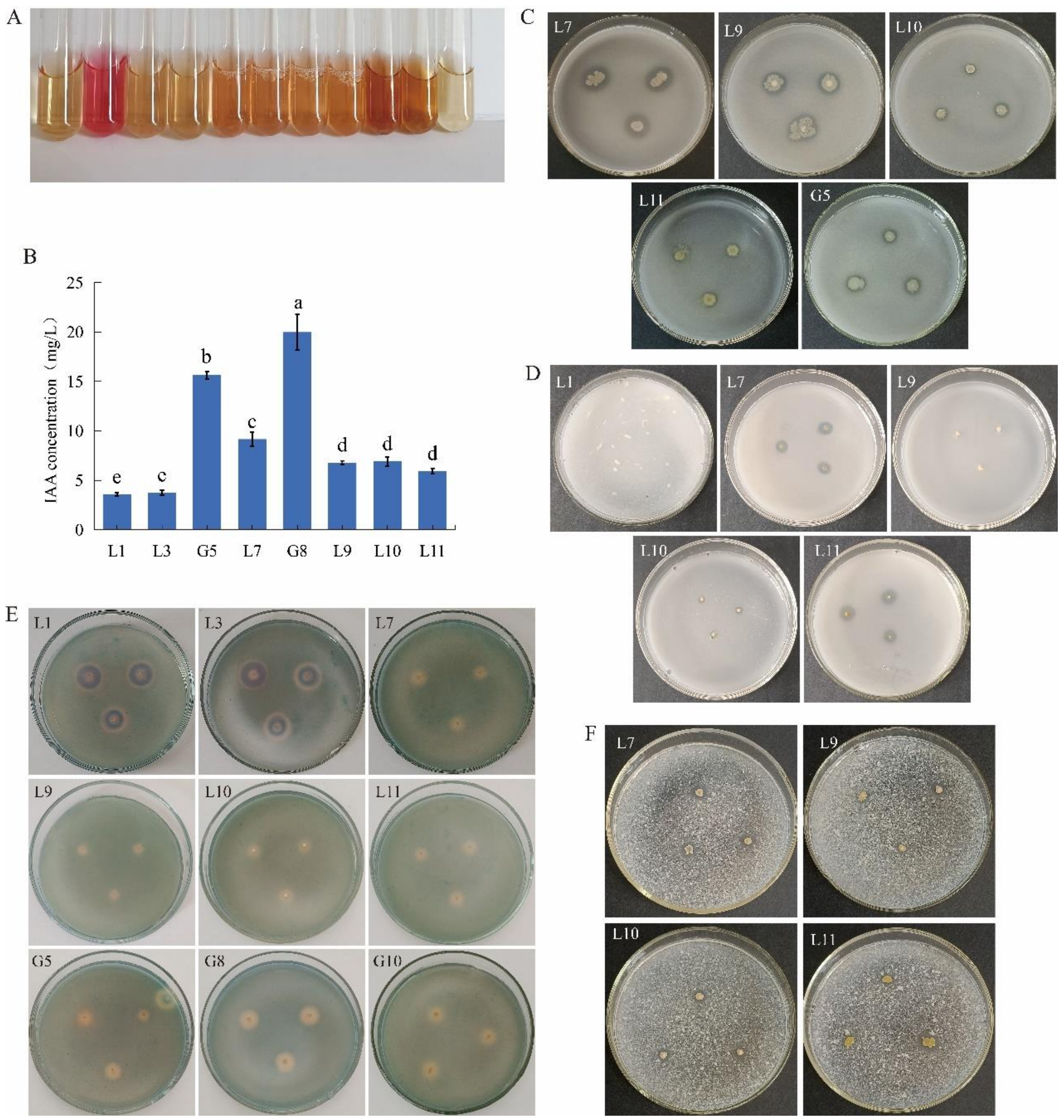

Figure S6. Determination of plant-growth-promoting abilities of bacterial strains. (A) Qualitative analysis of IAA production; from left to right: negative control, positive control, L1, L3, L7, L9, L10, L11, G5, G8, and G10. (B) Quantitative analysis of IAA production (different letters indicate significant differences among treatments by one-way ANOVA, $p < 0.05$). (C) Organic phosphate solubilization capacity. (D) Nitrogen fixation ability. (E) Siderophore production capacity. (F) Inorganic phosphate solubilization capacity.

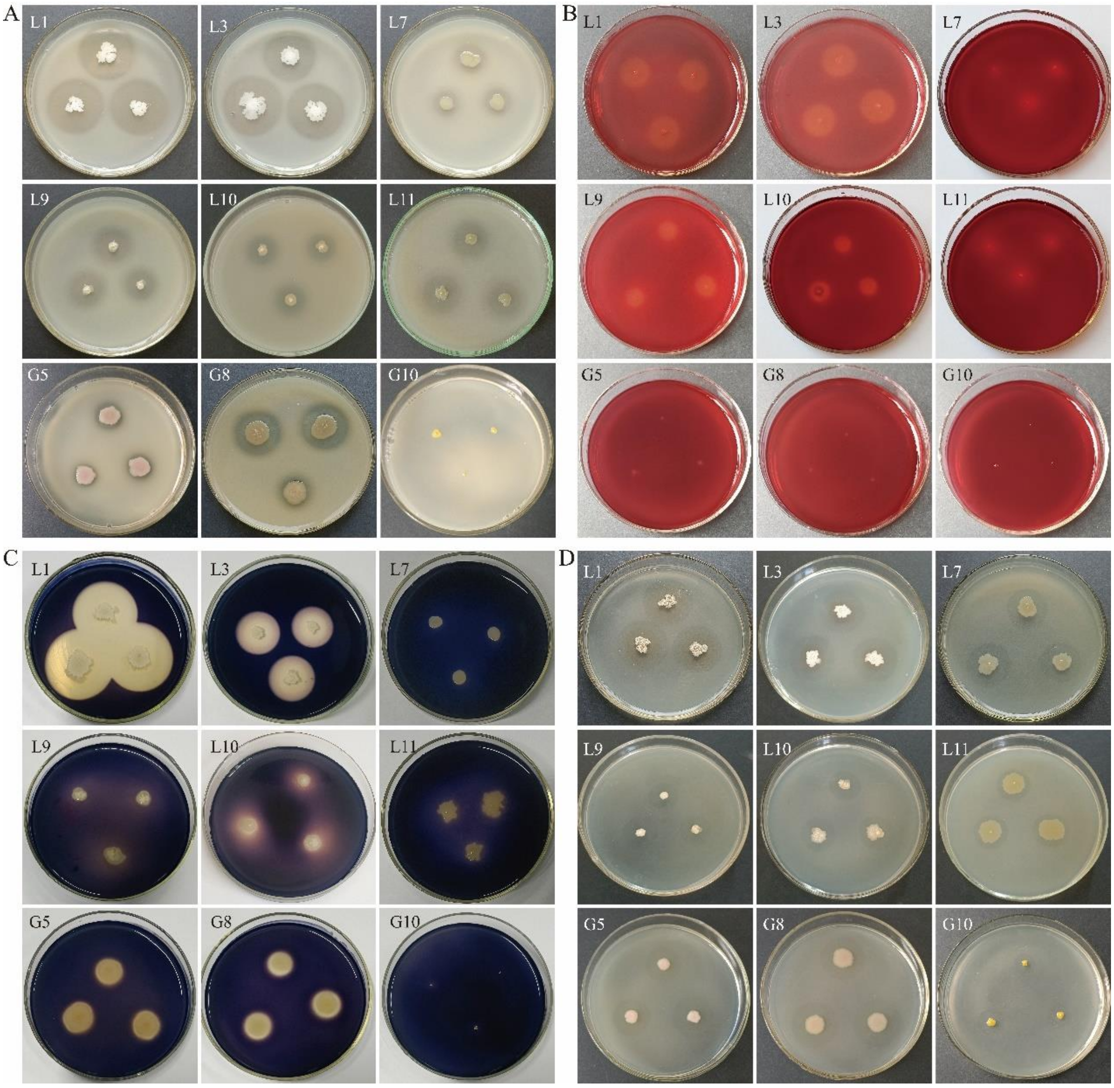

Figure S7. Enzyme production capacity of bacterial strains. (A) Protease production. (B) Cellulase production. (C) Amylase production. (D) Lipase production.

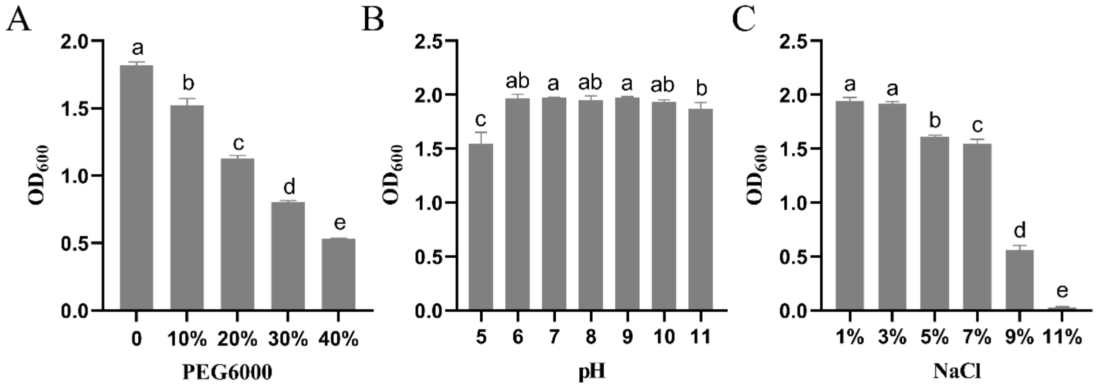

Figure S8. Growth of strain L7 under abiotic stresses of varying PEG6000 (10-40%), pH (5-11), and salt concentration (1-11%).

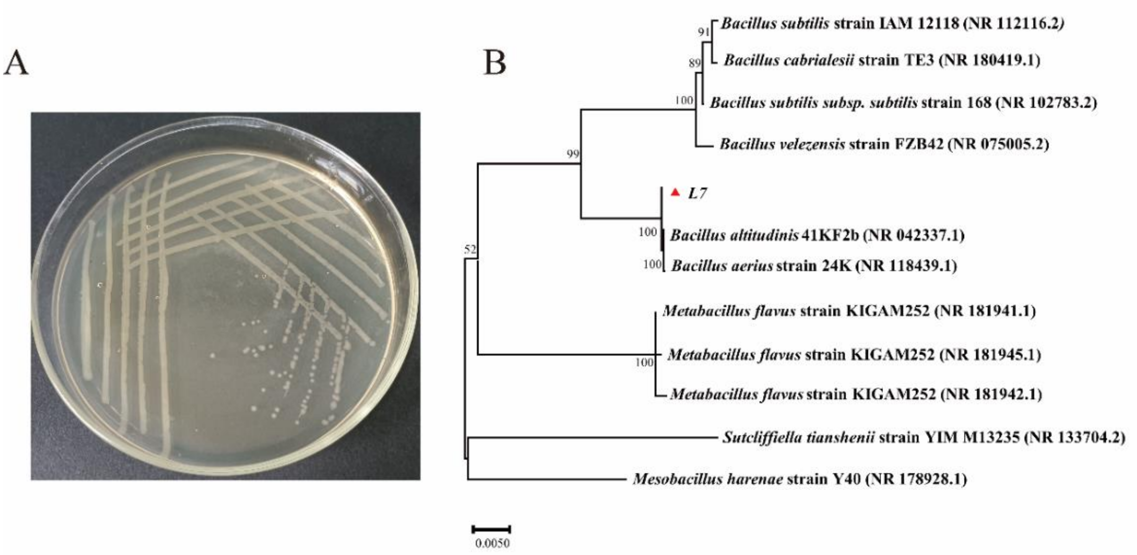

Figure S9. Morphological characteristics and phylogenetic analysis of strain L7. (A) Colony morphology of strain L7 on LB medium. (B) Phylogenetic tree of L7 constructed based on 16S rRNA gene sequences.

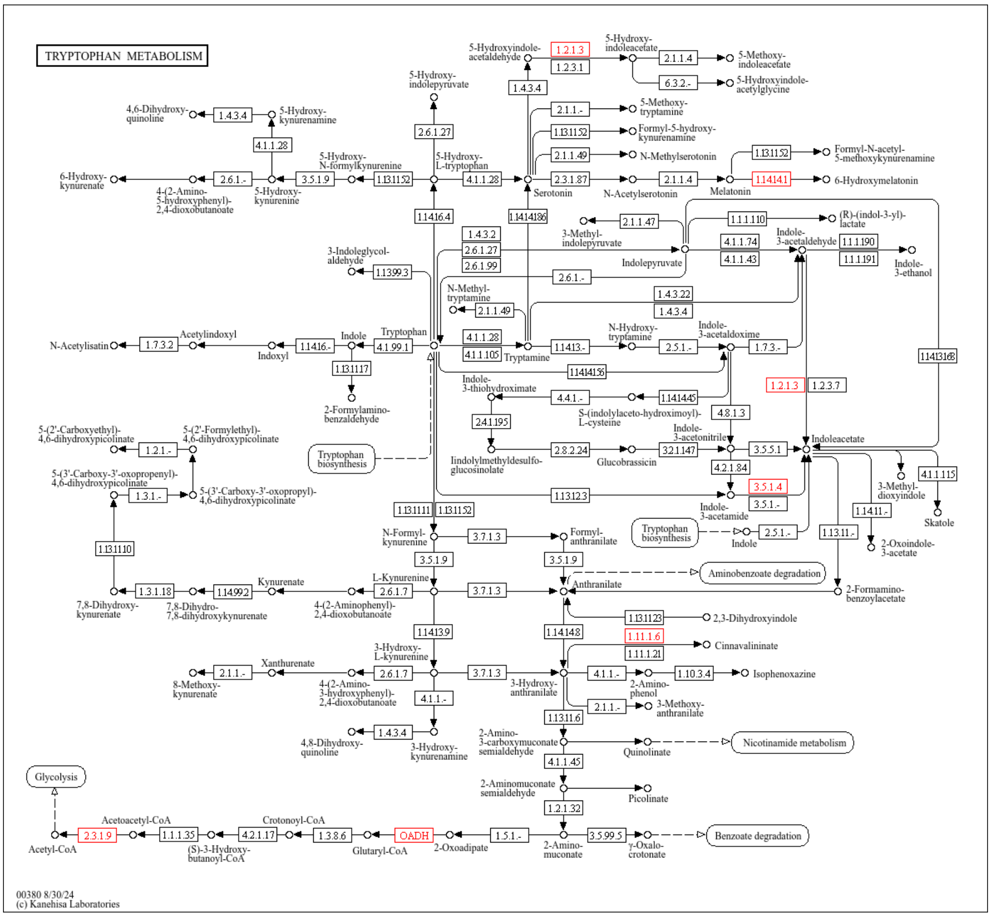

Figure S10. KEGG pathway of tryptophan metabolism in strain L7 (enzymes present in strain L7 are highlighted in red).

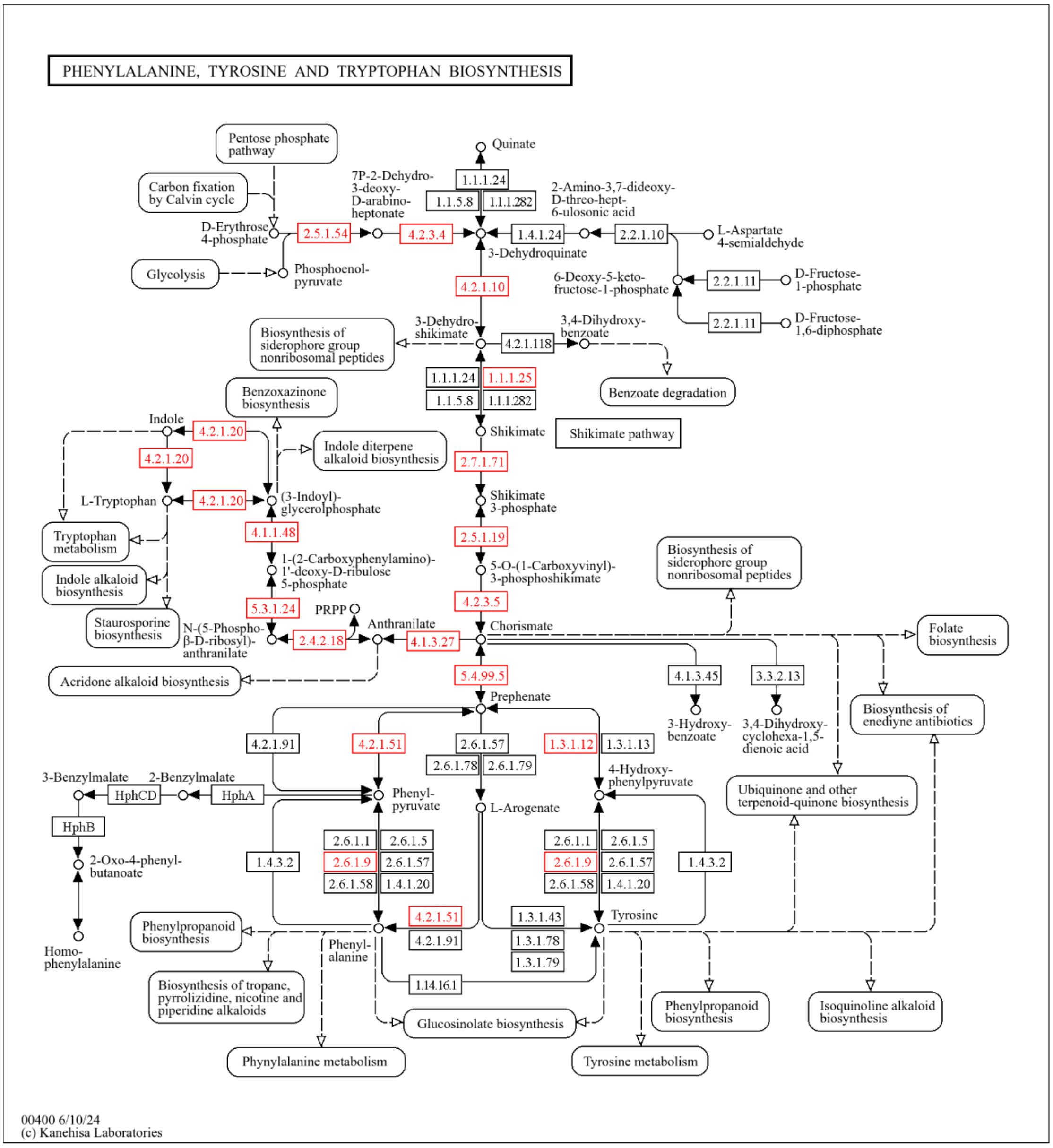

Figure S11. KEGG pathway of tryptophan biosynthesis in strain L7 (enzymes present in strain L7 are highlighted in red).

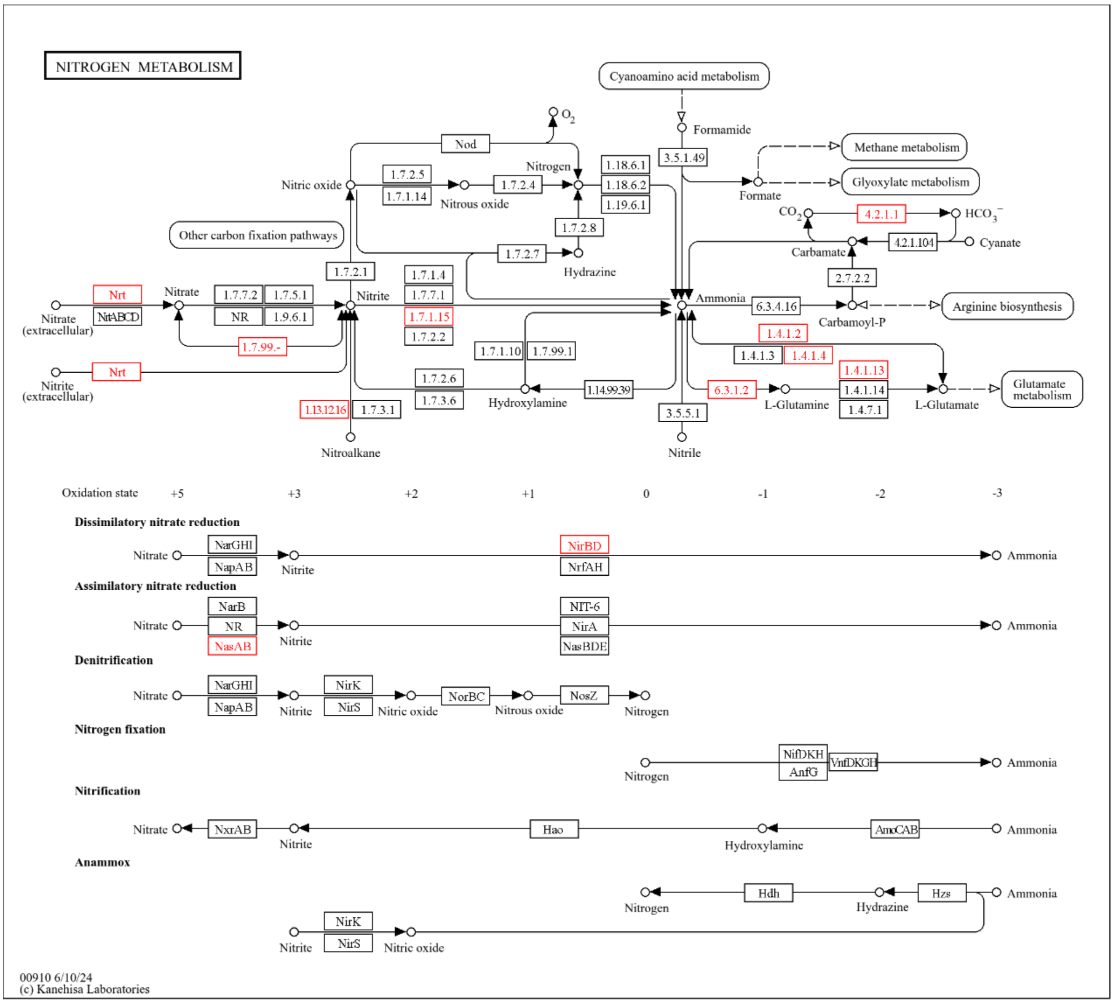

Figure S12. KEGG pathway associated with nitrogen metabolism in strain L7 (enzymes present in strain L7 are highlighted in red).

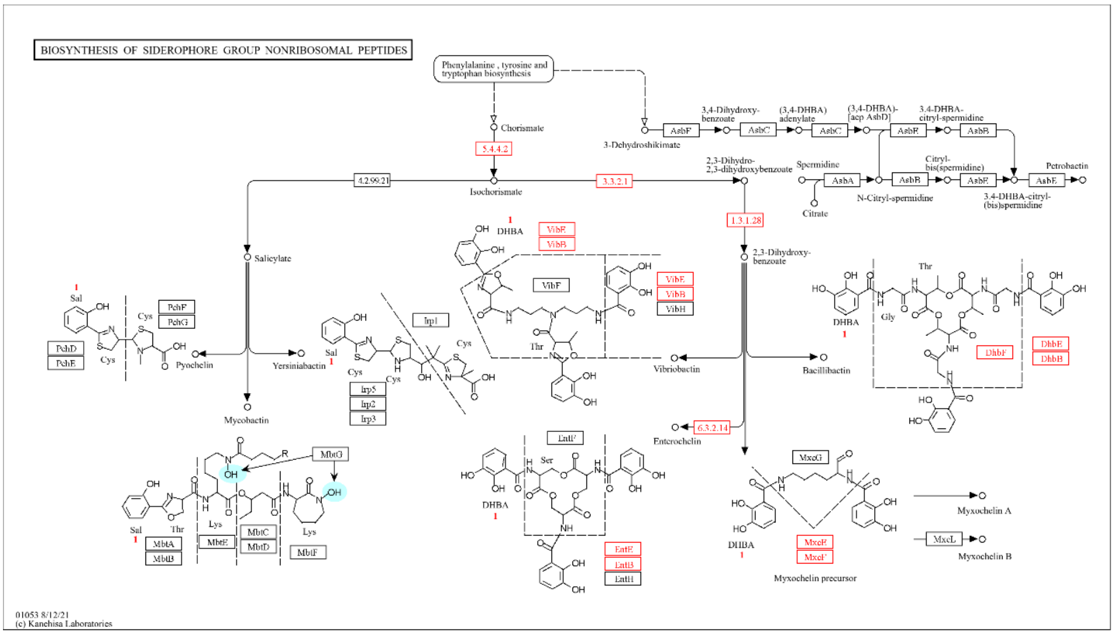

Figure S13. KEGG pathway associated with siderophore biosynthesis in strain L7 (enzymes present in strain L7 are highlighted in red).

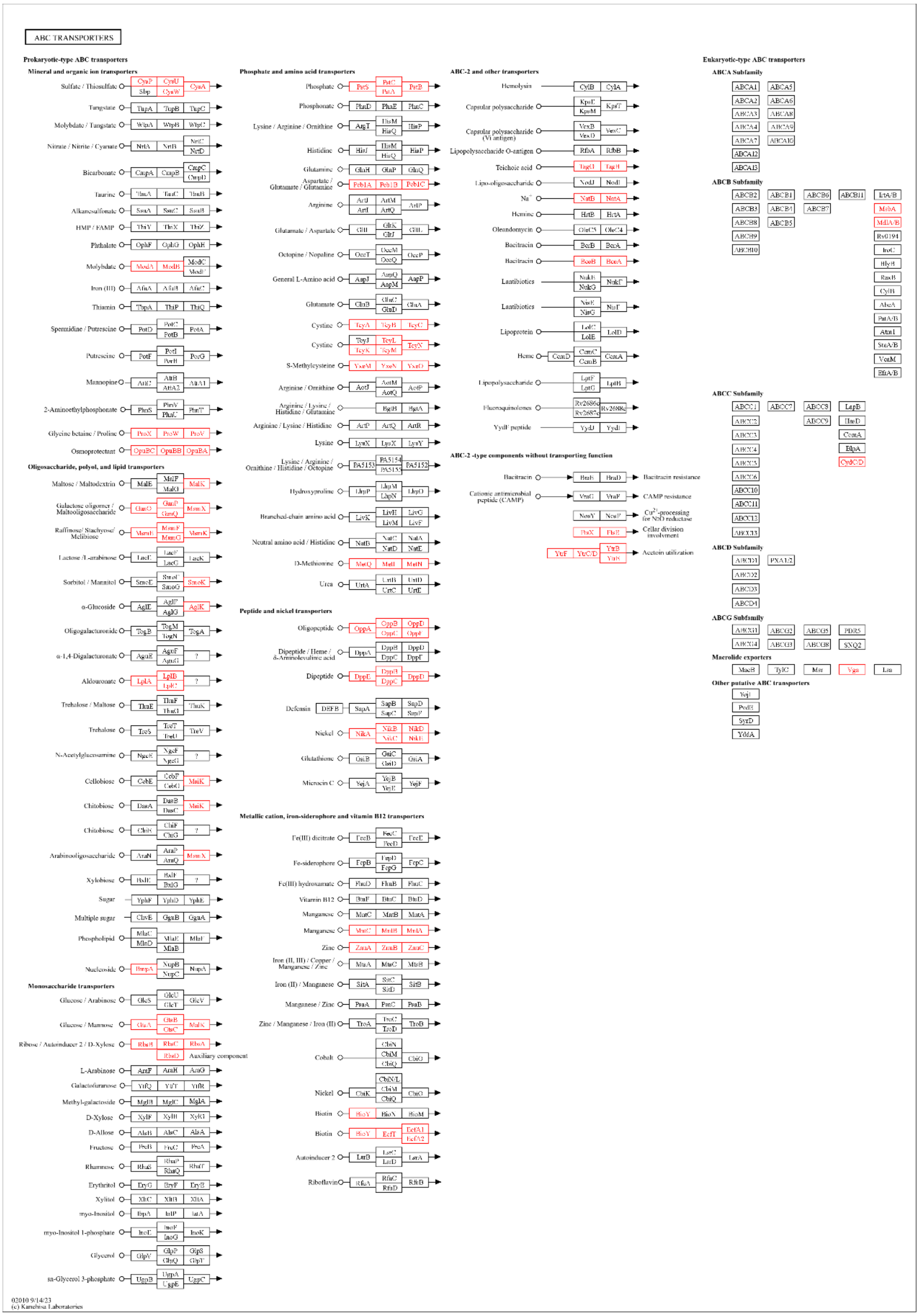

Figure S14. KEGG pathway of phosphate metabolism in strain L7 (enzymes present in strain L7 are highlighted in red).

Table S1. Summary statistics of alpha diversity indices for endophytic bacteria in *Stipagrostis pennata* seeds.

ID	Observed_species	Chao1	ACE	Shannon	Simpson
SPA	343 ± 58.39a	418.23 ± 34.71a	424.82 ± 26.12a	2.23 ± 0.97a	0.68 ± 0.24a
SPB	396.67 ± 80.45a	444.50 ± 86.36a	437.68 ± 75.03a	2.70 ± 0.78a	0.78 ± 0.15a
T-test *P*	0.41	0.66	0.80	0.55	0.59

Table S2. Genus-level identification of endophytic bacteria based on 16S rRNA gene sequencing.

Genus	Strain	Closest species match in GenBank	Identity (%)
Bacillus	L1	*Bacillus mojavensis* strain IFO 15718	100%
	L3	*Bacillus mojavensis* strain RS-1	100%
	L7	*Bacillus altitudinis* strain 41KF2b	100%
	L9	*Bacillus altitudinis* strain SAM1	100%
	L10	*Bacillus aerius* strain 24K	99.93%
	L11	*Bacillus subtilis* strain Op1-Ha4	100%
Peribacillus	G5	*Peribacillus frigoritolerans* strain DSM 8801	99.93%
	G8	*Peribacillus frigoritolerans* strain DSM 8801	99.93%
Microbacterium	G10	*Microbacterium oxydans* strain OdE4	100%

Table S3. Evaluation of plant-growth-promoting (PGP) functions of endophytic bacteria in *Stipagrostis pennata* seeds.

Strain	IAA production (mg/L)	Inorganic P solubilization (D/d)	organic P solubilization (D/d)	Siderophore production (D/d)	Nitrogen fixation
L1	3.57 ± 0.16	−	−	3.92 ± 0.64	+
L3	3.75 ± 0.26	−	−	4.06 ± 0.77	−
L7	9.16 ± 0.73	2.20 ± 0.13	2.26 ± 0.14	4.23 ± 0.38	+
L9	6.78 ± 0.19	1.91 ± 0.14	1.36 ± 0.01	3.12 ± 0.19	+
L10	6.91 ± 0.47	2.04 ± 0.11	1.52 ± 0.15	2.88 ± 0.29	+
L11	5.93 ± 0.28	2.09 ± 0.17	1.31 ± 0.03	3.64 ± 0.14	+
G5	15.65 ± 0.37	−	1.49 ± 0.01	3.75 ± 0.70	−
G8	19.99 ± 1.82	−	−	4.14 ± 0.04	−
G10	−	−	−	3.02 ± 0.27	−

Table S4. Hydrolytic enzyme production capacity of endophytic bacteria in *Stipagrostis pennata* seeds.

Strain	Amylase activity （D/d）	Protease activity (D/d)	Lipase activity (D/d)	Cellulase activity (D/d)
L1	2.84 ± 0.20	3.01 ± 0.15	3.05 ± 0.27	6.83 ± 1.07
L3	2.83 ± 0.03	2.55 ± 0.03	2.82 ± 0.41	6.25 ± 0.15
L7	−	1.78 ± 0.20	2.01 ± 0.09	3.66 ± 0.30
L9	1.18 ± 0.06	3.35 ± 0.35	3.40 ± 0.12	5.99 ± 0.71
L10	1.20 ± 0.02	2.83 ± 0.02	2.48 ± 0.54	4.16 ± 0.84
L11	−	2.73 ± 0.28	1.61 ± 0.09	3.54 ± 0.14
G5	1.29 ± 0.03	1.39 ± 0.11	2.73 ± 0.43	−
G8	1.63 ± 0.12	1.85 ± 0.31	2.32 ± 0.07	−
G10	−	−	3.13 ± 0.42	−

Table S5. Physiological and biochemical characteristics of strain L7.

Physiology and biochemistry test	Result	Physiology and biochemistry test	Result
Gram staining	+	Glucose as the sole carbon source	+
Methyl red test	+	Sucrose as the sole carbon source	+
V-P reaction	−	Mannitol as the sole carbon source	+
Gelatin liquefaction	−	Xylose as the sole carbon source	+
H_2S production	−	L-Arabinose as the sole carbon source	+
Catalase	+	Inositol as the sole carbon source	−

"+", Positive reaction; "−", Negative reaction.

Table S6. Genomic features of endophytic strain L7.

Attribute	L7
Genome size	3,682,572 bp
Gene Length	3,287,958 bp
GC Content	41.73%
Gene Number	3,880
Non-coding RNA (ncRNA)	83
tRNA	71
5s	8
16s	1
23s	1
Small RNA (sRNA)	2
Long terminal repeat (LTR)	133
DNA repeat elements (DNA)	44

Attribute	L7
Long interspersed nuclear elements (LINE)	46
Short interspersed nuclear elements (SINE)	8
GIs	4
Prophage	2
Genes assigned to NR	3,792
Genes assigned to Swiss-Prot	2,870
Genes assigned to COG	2,891
Genes assigned to KEGG	3,678
Genes assigned to CAZy	138
Genes assigned to Pfam	2,701
Genes assigned to TCDB	493
Genes assigned to GO	2,701

Table S7. Predicted genes associated with PGP and stress resistance functions in the genome of strain L7.

Characteristics	Gene name	Gene annotation	ID
Phosphate solubilization	*pstA*	phosphate transport system permease protein	K02038
	pstB	phosphate transport system ATP-binding protein	K02036
	pstC	phosphate transport system permease protein	K02037
	pstS	phosphate transport system substrate-binding protein	K02040
	phoB	alkaline phosphatase	K01077
	phoR	phosphate regulon sensor histidine kinase PhoR	K07636
	phoA	alkaline phosphatase	K01077
	appA	Oligopeptide-binding protein AppA	P42061
	pit	Probable low-affinity inorganic phosphate transporter	O34436
	phnA	protein PhnA	K06193
	phnB	PhnB protein	K04750
	phoE	uncharacterized phosphatase	K15640
	phoB1/phoP	alkaline phosphatase synthesis response regulator PhoP	K07658
	phoH/L	phosphate starvation-inducible protein PhoH and related proteins	K06217

Table S7. (continued)

Characteristics	Gene name	Gene annotation	ID
IAA Production	*trpA*	tryptophan synthase alpha chain	K01695
	trpB	tryptophan synthase beta chain	K01696
	trpF	phosphoribosylanthranilate isomerase	K01817
	trpC	indole-3-glycerol phosphate synthase	K01609
	trpD	anthranilate phosphoribosyltransferase	K00766
	trpE	anthranilate synthase component I	K01657
Siderophore	*fhuC*	Iron(3+)-hydroxamate import ATP-binding protein FhuC	P49938
	fhuG	Iron(3+)-hydroxamate import system permease protein FhuG	P49937
	fhuB	Iron(3+)-hydroxamate import system permease protein FhuB	P49936
	fhuD	Iron(3+)-hydroxamate-binding protein FhuD	P37580
	entA	2,3-dihydro-2,3-dihydroxybenzoate dehydrogenase	K00216
	entB/dhbB/vibB/ mxcF	bifunctional isochorismate lyase / aryl carrier protein	K01252
	entC	isochorismate synthase	K02361
	entE/dhbE/vibE/mxcE	2,3-dihydroxybenzoate-AMP ligase	K02363
	dhbF	nonribosomal peptide synthetase DhbF	K04780
Nitrogen fixation	*nifS*	Putative cysteine desulfurase NifS	P38033
	iscU/nifU	nitrogen fixation protein NifU and related proteins	K04488
	sufB	Fe-S cluster assembly protein SufB	K09014
	sufC	Fe-S cluster assembly ATP-binding protein	K09013
	sufD	Fe-S cluster assembly protein SufD	K09015
Nitrogen metabolism	*glnA*	glutamine synthetase	K01915
	narK/nasA/nrtP	MFS transporter, NNP family, nitrate/nitrite transporter	K02575
	nirB	nitrite reductase (NADH) large subunit	K00362
	nirD	nitrite reductase (NADH) small subunit	K00363
	norM	multidrug resistance protein, MATE family	K03327
	nos	nitric-oxide synthase, bacterial	K00491
	gudB/rocG	glutamate dehydrogenase	K00260
	gltB	glutamate synthase (NADPH) large chain	K00265
	gltC	LysR family transcriptional regulator, transcription activator of glutamate synthase operon	K09681

Table S7. (continued)

Characteristics	Gene name	Gene annotation	ID
Nitrogen metabolism	*gltD*	glutamate synthase (NADPH) small chain	K00266
Salt tolerance	*nhaC*	Na+:H+ antiporter, NhaC family	K03315
	mrp/mnh(A-G)	multicomponent Na+:H+ antiporter subunit	K05565-K05571
	trkA	trk system potassium uptake protein	K03499
Tolerant to both salt and drought	*proA*	glutamate-5-semialdehyde dehydrogenase	K00147
	proB	glutamate 5-kinase	K00931
	proC	pyrroline-5-carboxylate reductase	K00286
	ectB	diaminobutyrate-2-oxoglutarate transaminase	K00836
	betL/opuD	glycine betaine transporter	K05020
	opuA	osmoprotectant transport system ATP-binding protein	K05847
	opuBD	osmoprotectant transport system permease protein	K05846
	opuC	osmoprotectant transport system substrate-binding protein	K05845
	proV	glycine betaine/proline transport system ATP-binding protein	K02000
	proW	glycine betaine/proline transport system permease protein	K02001
	proX	glycine betaine/proline transport system substrate-binding protein	K02002
	dnaK	molecular chaperone DnaK	K04043
	dnaJ	molecular chaperone DnaJ	K03686
	HSP20	HSP20 family protein	K13993
	groES	chaperonin GroES	K04078
	groEL	chaperonin GroEL	K04077
